# Characteristics and outcomes of children initiated on high flow nasal cannula and continuous positive airway pressure at the emergency centre of a district hospital in South Africa

**DOI:** 10.1016/j.afjem.2025.100884

**Published:** 2025-06-16

**Authors:** Jessica Head, Andrew Redfern, Jana Hoole, Liezl Ulbrich, Refilwe More, Daniël J. van Hoving, Eric D. McCollum, Shubhada Hooli

**Affiliations:** a Department of Paediatrics, Khayelitsha Hospital, Cape Town, South Africa; b Department of Paediatrics & Child Health, Stellenbosch University, Cape Town, South Africa; c Paediatric Emergency and Ambulatory Unit, Tygerberg Hospital, Cape Town, South Africa; d Western Cape Government Emergency Medical Services, Cape Town, South Africa; e Human Sciences Research Council Internship Program, Cape Town, South Africa; f Division of Emergency Medicine, Faculty of Medicine and Health Sciences, Stellenbosch University, Cape Town, South Africa; g Health Systems Program, Department of International Health, Johns Hopkins Bloomberg School of Public Health, Baltimore, MD, USA; h Global Program in Pediatric Respiratory Sciences, Eudowood Division of Pediatric Respiratory Sciences, Department of Pediatrics, Johns Hopkins School of Medicine, Baltimore, MD, USA; i Department of Pediatrics, Division of Emergency Medicine, Baylor College of Medicine, Houston, TX, USA

**Keywords:** High flow nasal cannula, Continuous positive airway pressure, Child, Pneumonia, South Africa, Low- and middle-income countries

## Abstract

**Introduction:**

High-flow nasal cannula (HFNC) and continuous positive airway pressure delivered via a nasal interface (nCPAP) are increasingly used for paediatric emergency care in South Africa. In Cape Town, initiation of HFNC/nCPAP at a district hospital, in most instances, necessitates transfer to a paediatric high-care facility. We sought to describe the population of children initiated on HFNC/nCPAP and their short-term hospital outcomes post interfacility transfer.

**Methods:**

The authors conducted a one-year retrospective observational study between August 1st 2021, to July 31st^,^ 2022 of children initiated on HFNC or nCPAP in the emergency centre (EC) of Khayelitsha district Hospital and transferred by ambulance to Tygerberg Hospital paediatric emergency centre. Children were excluded from the study if they were <10 days or >13 years of age, if they had an advanced care plan that restricted the escalation of respiratory support or if their medical records were incomplete.

**Results:**

At Khayelitsha Hospital, 117 patients were initiated on HFNC (*n* = 58) or nCPAP (*n* = 59). Participants had a median age of 6.8 months. There were no major adverse events reported during inter-facility transfer. Respiratory support was weaned to low flow oxygen or room air within 24 h of transfer in 23.9 % and escalated in 9.4 %. During hospital stay 14.5 % were admitted to intensive care, 6.0 % ultimately required mechanical ventilation, and the in-hospital mortality rate was 1.7 %.

**Conclusion:**

Roughly a quarter of patients were weaned from respiratory support within 24 h of transfer. Short term outcomes were good overall, demonstrating safe interfacility transfer and low mortality. Further research is needed to inform practice on best use of HFNC and nCPAP in the emergency care of children presenting with acute respiratory illness in South Africa.

## African relevance

• HFNC & nCPAP are increasingly used for children with acute respiratory illness.
• Emergency Centre initiation of HFNC/nCPAP and inter-facility transfer is feasible and safe.
• Patient selection is critical due to the cost and resource implications.
• Context specific & pragmatic guidelines are needed.

## Introduction

**Fig. 1:**
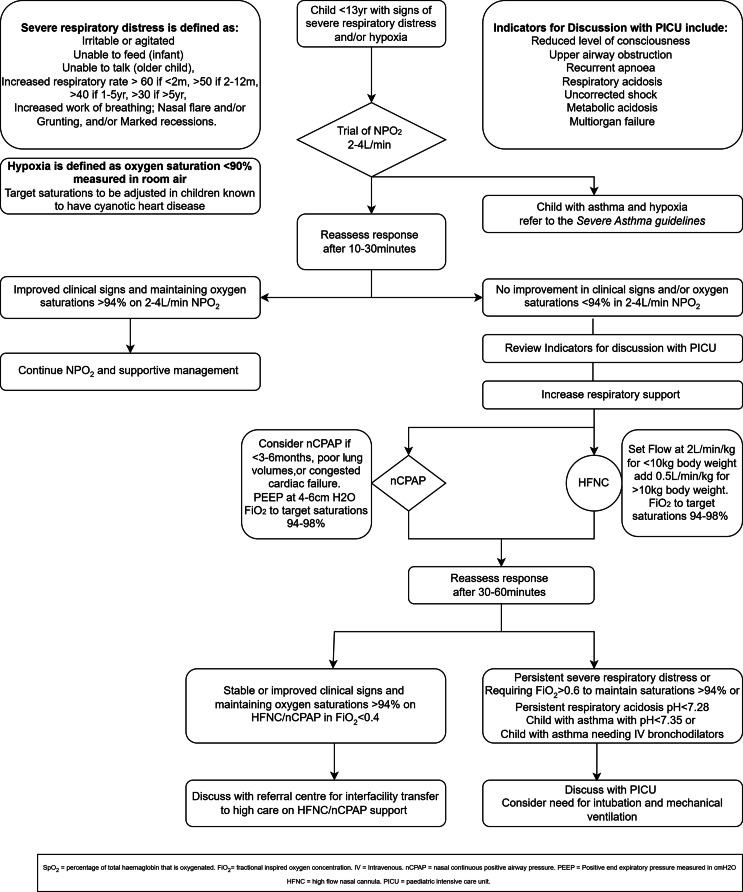
Flow Chart Summarising Local Guidelines for Initiation of HFNC and nCPAP for Children with Respiratory Distress and/or Hypoxemia.

Acute lower respiratory tract infections (ALRIs) are a leading cause of death globally and the primary cause of death of children under 5 years old in the Western Cape, South Africa [1,2]. Children hospitalised with respiratory failure may benefit from respiratory support via heated, humidified high-flow nasal cannula (HFNC) or continuous positive airway pressure delivered via nasal interface (nCPAP) to avoid further deterioration leading to mechanical ventilation. During the COVID-19 pandemic, HFNC and nCPAP became available at many district hospitals in South Africa. A recent study in the United Kingdom suggested HFNC was non-inferior to nCPAP when used as a first mode of respiratory support for infants in intensive care units [3]. Though HFNC is integrated into local guidelines (Fig. 1) there are few reports on its use in our region and the most appropriate use in resource constrained settings remains uncertain [4,5].

HFNC has a strong safety profile, but the optimal timing for its initiation remains unclear [6] It delivers airflow that surpasses the patient’s inspiratory demand, effectively clearing exhaled carbon dioxide (CO₂) from non-gas-exchanging airways [7] This ensures inhaled air contains minimal CO₂, referred to as dead space washout, compared to low-flow oxygen. Humidification provided by HFNC increases mucous clearance, maintains mucociliary function, and decreases airway inflammation, which aides lung function [8] While HFNC may deliver some positive end-expiratory pressure, evidence is scant [9] One advantage of HFNC is that it allows for safe oral feeding, which may help prevent further illness-related weight loss in undernourished children [10] In the United States, in part due to a lack of evidence on the optimal time for initiation, overuse is well-documented [11] In that setting many children receiving HFNC in non-intensive care settings might be safely managed on low-flow oxygen instead [12] nCPAP delivers constant positive airway pressure improving lung volumes and compliance and decreasing work of breathing. By increasing mean airway pressure it recruits atelectatic lung, correcting ventilation-perfusion mismatch and improving gas exchange [13] Use of nCPAP in children living in resource-constrained settings is not well established. Four randomised controlled trials in Bangladesh, Ghana, Malawi, and Ethiopia aimed to address this. In a rural district hospital in Ghana, nCPAP improved outcomes for some children under 12 months old [14], while in Malawi, mortality in a hospital with minimal physician supervision increased [15] In Ethiopia physician supervised nCPAP in children under five years old at district hospitals led to fewer treatment failures and deaths compared with low-flow oxygen [16] The Bangladeshi trial demonstrated safety but found no difference in clinical outcomes in children initiated on nCPAP vs HFNC. However, the study was inconclusive as it was suspended due to increased mortality in the low-flow group [17]

We aimed to describe the characteristics of children initiated on HFNC or nCPAP at a district hospital, and their short-term outcomes following interfacility transfer to a referral hospital in Cape Town.

## Methods

This study was conducted in accordance with the Strengthening the Reporting of Observational Studies in Epidemiology (STROBE) guidelines. This retrospective observational study analysed emergency centre, inter-facility transfer, and hospital inpatient data. Clinical care practices followed South Africa Department of Health recommendations and local protocols (Fig. 1). We screened children for inclusion who were evaluated in the Khayelitsha Hospital (KH) Emergency Centre between August 1, 2021, and July 31, 2022. Children aged ten days to 12 years old initiated on HFNC or nCPAP and transferred to Tygerberg Hospital (TH) Paediatric Emergency Centre by the Western Cape Emergency Medical Services (EMS) Specialised Paediatric Retrieval and Neonatal Transfer Team (SPRINTT) were included. KH exclusively contracts with SPRINTT for the interfacility transfer of children on nCPAP or HFNC. We excluded children who were transferred to or from an inpatient unit, had a palliative care plan that limited escalation of respiratory support, or had incomplete hospital records. Eligible subjects were screened at EC encounters and could be enrolled multiple times.

The study sites were Tygerberg Hospital (TH), a regional referral centre for Khayelitsha and a quaternary care facility in Cape Town, and Khayelitsha Hospital (KH), a large district hospital 26 km away. Khayelitsha, a peri‑urban community of about 1.5 million people, has around 40 % of its residents under 13 years old [18] Many residents live in informal housing with limited water, sanitation, and electricity access. KH has a large emergency care facility that treats around 10,000 children each year. The Emergency Centre is staffed by junior doctors with two to three years of experience, supported by specialist emergency care physicians. KH has a general paediatric inpatient ward that lacks a high-care unit. Children initiated on HFNC or nCPAP are transferred to TH by SPRINTT retrieval service, which includes paramedics trained to manage paediatric respiratory support.

The types of equipment used for respiratory support differ between KH, TH, and SPRINTT. All groups utilize the *Airvo* High Flow System (Fisher & Paykel), SPRINTT make use of a UPS power supply for Airvo. nCPAP is delivered by a ventilator (Hamilton C1) via a RAM cannula (Medin) at KH and SPRINTT, whereas TH employs a custom-made ‘simple’ CPAP device. This comprises of a MR850 humidifier (Fisher & Paykel) with a flow-driven CPAP generator, nasal prongs (Intersurgical), and an analogue manometer. Local guidelines (Fig. 1) do not make strong recommendations in terms of which mode of respiratory support to use initially, as cost and availability of consumables necessitate a pragmatic approach. However, local practice preference is to initiate nCPAP as first choice in infants <3–6 months.

The study team queried a SPRINTT database to generate a list of patients who potentially met study inclusion criteria. Records of transfers between KH and TH during the study period were interrogated and search terms were Khayelitsha, Tygerberg, High Flow, HFNC, NIV, and CPAP. The inclusion list was cross-referenced with the Hospital and Emergency Centre Tracking Information System (HECTIS), which records all dispositions from the KH Emergency Centre, to ensure no cases were missed. The KH Electronic Content Management (ECM) record was reviewed by at least two study team members (JH, AR, JH, SH) to ensure potential cases met study inclusion/exclusion criteria. A final list of cases, including assigned identifier, date of encounter, medical record number, sex, and date of birth, was entered into a REDCap database [19] Study team members (JH, AR, JH, RM, SH) extracted data from KH and TH ECM records and entered these into the REDCap database.

Variables were extracted from notes in the medical records. Hypoxaemia was defined as peripheral oxygen saturation (SpO2) below 90 % on room air. The authors defined age-adjusted tachycardia and tachypnea using established age-adjusted nomograms and WHO criteria [20,21], Children were triaged according to the validated Paediatric South African Triage Scale (pSATS) [22] Urgency is first categorized as Emergency (Red), Very urgent (Orange), Urgent (Yellow), or Not urgent (Green) based on clinical discriminators. Then the triage nurse assesses the child’s mobility, level of alertness, heart rate, respiratory rate, and temperature. The Triage Early Warning Score (TEWS) is a composite score of these factors, also categorised by colour. Whichever colour category is more urgent is the final assigned triage category. Children were classified as living with Human Immunodeficiency Virus (HIV) if they were known to be HIV infected or newly diagnosed during admission. HIV diagnosis was determined by rapid antibody and confirmatory serum ELISA testing. Children under 12 months old were classified as HIV-exposed and uninfected if their mother was living with HIV but HIV tests on the child were negative.

The authors sought to describe demographic and clinical characteristics, the primary outcome of interest, and the hospital outcome of the study population in frequencies and proportions. Differences between groups of children transferred on HFNC and nCPAP were compared using Student’s *t*-test for normally distributed data, Mann-Whitney U for non-parametric data, and Chi-squared and Fisher’s Exact tests for proportions. Stata® software version 14.2 was used to conduct analyses [23]

Ethical approval was provided by Stellenbosch University Human Research Ethics Committee (Project ID 24,820) and the Institutional Review Board of Baylor College of Medicine (Protocol H-52,145). As the study involved minimal risk and was retrospective, the ethics committee granted a waiver of informed consent.

## Results

We identified 179 patients from the databases. Of these, five were duplicates, 36 did not meet inclusion criteria, and 21 had missing records; the final dataset contained 117 encounters. A similar number of children were initiated on HFNC (*n* = 59) and nCPAP (*n* = 58) with an overall median age of 6.8 months. Notably, the nCPAP group was significantly younger, 2.8 (IQR 1.7–6.9) vs 12.5 months (IQR 6.4–24.5) for HFNC (*p* < 0.001). Of the children on nCPAP, 50/58 (86 %) were <12 months, compared to only 25/59 (42 %) of the HFNC group (Table 1). Children over 5 years old comprised 7/117 (6.0 %) of the study population. 38/117 (33 %) children were exposed to HIV, 5 were known to be infected. There was no difference in the number of nCPAP and HFNC cases with a weight-for-age z-score ≤ −3, consistent with being severely underweight (15.4 %). Other co-morbidities and pre-existing conditions were not commonly documented in the medical record. Most patients (82.9 %) were diagnosed with pneumonia and almost all received antibiotics (94.9 %).

**Table 1:** Patient Characteristics and Outcomes by Initial Mode of Support.

Characteristics	HFNC (*n* = 59)	nCPAP (*n* = 58)	P value
Age in months, median (IQR)	12.5 (6.4 – 24.5)	2.8 (1.7 – 6.9)	<0.001
Age groups, n (%) 10 days – 1 months 2 – 11 months 12–59 months 5–10 yr Over 10 yr	0 25 (42.4) 28 (47.5) 6 (10.2) 0 (0)	21 (36.2) 29 (50.0) 7 (12.1) 1 (1.7) 0 (0)	<0.001
Male, n (%) Female, n (%)	31 (52.5) 28 (47.5)	35 (60.3) 23 (39.7)	0.395
HIV unexposed, uninfected, n (%) HIV exposed, uninfected, n (%) HIV infected, n (%) HIV status unknown, n (%)	36 (61.0) 15 (25.4) 1 (1.7) 7 (11.9)	36 (62.1) 18 (31.0) 4 (6.9) 0 (0)	0.020
Pulmonary TB not diagnosed, n (%) Previous pulmonary TB disease, n (%) Probable/confirmed pulmonary TB disease, n (%)	53 (89.8) 1 (1.7) 3 (5.1)	55 (94.8) 1 (1.7) 2 (3.4)	0.757
Tachypnoea on arrival, n (%)	41 (69.5)	32 (55.2)	0.084
Tachycardia on arrival n (%)	49 (83.1)	48 (82.8)	0.584
SpO_2_ % on room air on arrival, median (IQR) SpO_2_ > 90 %, n (%) SpO_2_ < 90 %, n (%) Missing, n (%)	88 (85–94) 13 (22.0) 33 (55.9) 13 (22.0)	89 (84–93) 26 (44.8) 29 (50.0) 3 (5.2)	0.184 0.051
Trial of 2–4L/min low flow oxygen, n (%)	53 (89.8 %)	53 (91.4 %)	0.774

**Outcomes**			
Weaned off HFNC/CPAP by 24 h, n (%)	18 (30.5)	10 (17.2)	0.093
Hospital length of stay in days, median (IQR)	5 [3–7]	6.5 (4–10.5)	0.038
Admitted to intensive care unit, n (%)	5 (8.5)	12 (20.7)	0.050
Required invasive ventilation, n (%)	1 (1.7)	6 (10.3)	0.061
Hospital deaths, n (%)	1	1	1.000

SpO_2_: Peripheral arterial oxyhaemoglobin saturation as measured with pulse oximeter.

TB: Tuberculosis.

HIV: Human Immunodeficiency Virus.

Most children were triaged with high acuity, 51.3 % emergency and 30.0 % very urgent according to pSATS [22] There were no statistical differences in acuity between those initiated on nCPAP and HFNC. However, it should be noted that SpO_2_ was missing more frequently on arrival amongst children on HFNC (22 %) vs nCPAP (5 %). Nearly all children (91.5 %) received a trial of supplemental low-flow oxygen prior to initiation of HFNC/CPAP.

**Fig. 2:**
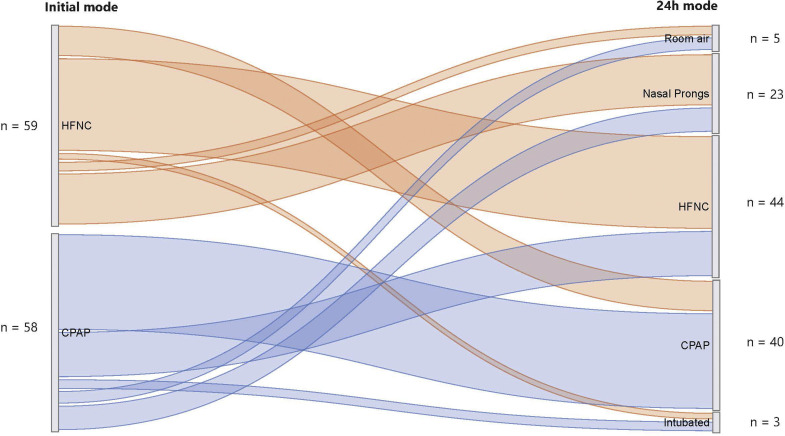
Changes in mode of respiratory support 24 h after initiation.

SPRINTT did not report any major adverse events, such as cardiac arrest, death or need for intubation, during inter-facility transfer. Following the transfer, the mode of respiratory support was changed within 24 h in 48/117 (41.0 %) cases, with 9/59 (15 %) of HFNC cases escalated to nCPAP and 14/58 (24 %) of nCPAP cases switched to HFNC (Fig. 2). Of cases weaned off HFNC or nCPAP 5/28 (18 %) were transitioned directly to room air, most were on low-flow oxygen delivered by nasal prongs (23/28) within 24 h of arrival at TH. There were no significant differences in age, initial mode of respiratory support, baseline triage category, or frequency of tachypnoea at presentation between children weaned off nCPAP or HFNC within 24 h and those who either remained on nCPAP or HFNC or required invasive ventilation (Table 2). However, children who were not hypoxaemic in room air on arrival at KH were more frequently weaned off HFNC/nCPAP (*p* = 0.045). Those with current or previous pulmonary TB were less frequently weaned off within 24 h (*p* = 0.039). Overall, the hospital length of stay was 1.5 days longer for the nCPAP group (6.5 vs 5 days, *p* = 0.038). During the hospital stay, 17/117 (14.5 %) of children were admitted to PICU, 71 % (12/17) of whom were initially on nCPAP (*p* = 0.05). Ultimately, 6.0 % (*n* = 7) of children required invasive ventilation, and two died (1.7 %).

**Table 2:** Characteristics and outcomes of children weaned off HFNC/nCPAP within 24 h and those who remained on HFNC/nCPAP or required invasive ventilation by 24 h.

Characteristics	Weaned off within 24 h (*N* = 28)	Remained on or required invasive ventilation by 24 h (*N* = 89)	p-value
Initial mode of support HFNC, n (%) nCPAP, n (%)	18 (64) 10 (36)	41 (46) 48 (54)	0.093
Age in months, median (IQR)	7 (3, 17)	6 (3, 15)	0.796
Male, n (%) Female, n (%)	14 (67) 12 (43)	61 (56.2) 39 (43.8)	0.929
HIV unexposed, uninfected, n (%) HIV exposed, uninfected, n (%) HIV-infected, n (%) HIV status unknown, n (%)	16 (57.1) 10 (35.7) 0 (0) 2 (7.1)	56 (62.9) 23 (25.8) 5 (5.6) 5 (5.6)	0.474
Pulmonary TB not diagnosed, n (%) Previous Pulmonary TB disease, n (%) Probable/confirmed Pulmonary TB disease, n (%)	26 (92.9) 0 (0) 0 (0)	82 (92.1) 2 (2.3) 5 (5.6)	0.039
Triage Early Warning Score on arrival, median (IQR)	6 (3,8)	6 (5,9)	0.089
Tachypnoea on arrival, n (%)	14 (50.0)	59 (66.3)	0.104
Tachycardia on arrival, n (%)	24 (85.7)	73 (82.0)	0.923
SpO_2_ % in room air on arrival, median, (IQR) SpO_2_ <90 %, n (%) SpO_2_ ≥90 %, n (%) Missing, n (%)	90 (88,93) 10 (35.7) 13 (46.4) 5 (17.9)	88 (84,92) 52 (58.4) 26 (29.2) 11 (12.3)	0.168 0.076 0.045
Arrived on low flow oxygen, n (%)	4 (14.3)	31 (34.8)	0.051

**Outcomes**			
Hospital Length of stay in days, median (IQR)	4 (2,8)	6 (4,10)	0.007
Admitted to intensive care unit, n (%)	1 (3.6)	16 (18.0)	0.064
Required invasive ventilation, n (%)	0	7 (7.9)	0.346
PICU Length of stay in days, median (IQR)	3	3 (2,11)	<0.001
Deaths, n (%)	0	2 (2.3)	1.000

SpO_2_: Peripheral arterial oxyhaemoglobin saturation.

TB: Tuberculosis.

HIV: Human Immunodeficiency Virus.

## Discussion

We described the population of children who presented to care at the emergency centre of KH initiated on either HFNC or nCPAP and their short-term outcomes following interfacility transfer to TH. Within 24 h of transfer, 23.9 % of patients were weaned to room air or low-flow oxygen. Only one required re-escalation of respiratory support during their hospital stay. There were no deaths during transport or within 24 h of arrival, and overall, a low mortality rate of 1.7 %. Most of the study population had a discharge diagnosis of pneumonia.

Evidence for choosing between HFNC or nCPAP as the first mode of support for children who fail low-flow oxygen therapy is limited. A UK study conducted in intensive care units with low patient-to-nurse ratios suggest non-inferiority between the two [3] At KH, physicians initiated HFNC or nCPAP with equal frequency, with younger infants more often on nCPAP. This aligns with local guidelines recommending nCPAP for respiratory failure in infants < 3–6 months (Fig. 1) and may be a pragmatic decision based on availability of consumables. Children initiated on nCPAP had longer hospital stays and more PICU admissions. This likely reflects its use in younger children, who have higher acute lower respiratory illness related mortality. Alternatively, variability in pressure delivery with the flow-regulated nCPAP at TH may play a role as the actual pressure delivered to the patient is less precisely controlled and may be less than the targeted 4–6 cmH_2_O. Further research is needed to determine the best support for children who fail low-flow oxygen therapy, considering underlying medical conditions, cost-effectiveness, and feasibility in this setting.

Comparing the study site EC experience with HFNC/nCPAP at other institutions is difficult due to limited literature. Few reports exist on HFNC in Africa, with none directly comparing it to nCPAP [24,25] The best comparison would be the implementation of HFNC at a nearby regional hospital in Cape Town,where following implementation of a program in 2015 to retain most patients on HFNC the proportion of patients receiving HFNC or being transferred rose from 6.5 % to 14 % [26] Over time, there were reductions in intubation and transfer rates [27] This underscores that the full impact of HFNC implementation at district hospitals in the region may take several years to become evident. In the same study, adverse events were reported in 7 out of 90 transfers, in contrast to this study which reported no major adverse events. Four of the 7 patients in whom adverse events occurred were intubated and two were transferred on rebreather masks. This study did not include intubated patients or patients not transferred on HFNC/nCPAP. Whilst adverse events may have been underreported, our lack of serious adverse events during interfacility transfer may also reflect the positive impact of the dedicated SPRINTT service, initiated in 2016. Our results are encouraging and support previous reported low frequency of serious adverse events during interfacility transfers of children on HFNC/nCPAP in the Westen Cape [26] This suggests that interfacility transfers, of under an hour by road, of children on HFNC/nCPAP may be safely performed by specialised ambulance teams in South Africa.

Changes in support modes were common within 24 h at TH. In the prior study, 24 % of children on HFNC were later escalated to nCPAP or required invasive ventilation [26] In our study, 17 % of children on HFNC had respiratory support escalated and 24 % of nCPAP-initiated children were switched to HFNC. Switching between modes of support incurs significant costs due to consumables which, in our setting, are typically more expensive for HFNC. Despite HFNC's acceptance in South Africa, robust cost analysis is lacking. Reasons for changing from nCPAP to HFNC may be valid such as the need to feed orally and patient comfort [22] Our study did not explore the reasons for changing support modes; local guidelines currently suggest escalation from HFNC to nCPAP in children not responding to HFNC, conversely clinicians might be using HFNC as a step-down from nCPAP. Alternatively, the reasons may be more pragmatic such as availability of devices and consumables and ease of HFNC set up compared to nCPAP.

The data is insufficient to identify factors influencing respiratory support use. Local guidelines recommend escalation for SpO_2_ < 94 % or severe respiratory distress after a trial of low-flow oxygen therapy (Fig. 1). Without pulse oximetry, a raised respiratory rate and increased work of breathing may indicate hypoxaemia and the need for oxygen therapy [28] However, vital signs did not appear to drive the escalation of respiratory support from low-flow oxygen. It should be noted that a significant proportion of children did not have their oxygen saturation on room air recorded prior to initiation of HFNC/nCPAP, this may happen if the child had been transferred in from a primary health care facility and arrived on low flow oxygen with signs of severe distress. Of the children who did have oxygen saturation documented prior to initiation of HFNC/nCPAP, half were not hypoxaemic, additionally over one-third of children started on HFNC/nCPAP were not tachypnoeic on initial assessment. Other clinical features, such as increased work of breathing, mental status, or an impression of “tiring” may have influenced the decision to initiate HFNC or nCPAP. We attempted to infer this data from the medical record but did not achieve reasonable interrater reliability. Similarly, the factors driving weaning of support could not be reliably derived from our data. A small number (*n* = 28) were weaned off support within 24 h of transfer. These children were less frequently hypoxaemic on arrival. We are unable to determine from this study if these cases represent a trend towards overuse, following similar patterns observed in the United States [11] Given that 24 % of cases were weaned from support within 24 h, it is worthwhile to explore if this could be done safely at the district-level hospital, saving the costs of inter-facility transfer and high-care bed occupancy [27] Implementation of a standardised care pathway for initiating and weaning off support may further help reduce the time on respiratory support, oxygen therapy, and hospital length of stay.

As this was a retrospective study with data extraction from multiple handwritten and electronic sources, the greatest limitation was data quality. The authors were unable to evaluate 11.7 % of the total number of potential cases identified by the SPRINTT databases for study inclusion/exclusion criteria due to missing records, which may have led to selection bias. Malnutrition is a known pneumonia-related mortality risk factor [27], and as prematurity and patient length/height was infrequently documented, the authors were unable to report accurate weight-for-age/weight-for-height Z scores. Nutritional status and prematurity likely influenced clinical decision-making to initiate HFNC or nCPAP support. It is also possible that some children had other undocumented comorbidities. Most were diagnosed with pneumonia rather than viral bronchiolitis. It is beyond the scope of this study to evaluate how the diagnosis of pneumonia was made.

Because of inconsistencies in the way HFNC flow rate, nCPAP pressure, and the fraction of oxygen are documented, we were unable to report how these factors changed as care was weaned. The nCPAP devices KH and TH used are different as the custom nCPAP used at TH does not have sensors to maintain the constant level of pressure throughout the respiratory cycle and requires maintenance of adequate prong positioning. Thus, it is difficult to ascertain the precise amount of PEEP delivered with this device, and it may provide lower PEEP compared to the Hamilton C1 used at KH. This may have contributed to the progression of the disease or hospital length of stay in patients managed on nCPAP at TH. Some children may have waited many hours for transfer on the Hamilton C1 nCPAP, total time on support may have been longer for some, which may have influenced readiness for weaning off nCPAP once at TH. Finally, this study was conducted at a single district hospital in the Western Cape, limiting its generalizability.

## Conclusions

HFNC and nCPAP are two respiratory support modalities increasingly utilised in district-level health facilities in South Africa. The authors observations support that these can be safely initiated in district hospitals with minimal interfacility transfer adverse events. These patients experience low overall mortality frequency. Further prospective research is needed to determine which patients would be best managed on low-flow oxygen, HFNC, and nCPAP with the goal to refine initiation, escalation and weaning practices.

## Dissemination of results

Results from this study have been presented at the South African Paediatric Association conference 2023 and at the African Federation of Emergency Medicine conference 2024. Results have been shared with colleagues at both study sites and displayed by poster in the KH Emergency Centre and at the Stellenbosch University Faculty of Medicine and Health Sciences annual research day.

## Author contributions

JH: Conceptualisation, Project administration, Data curation, Visualization, Writing-original draft; AR: Conceptualisation, Data curation, Writing-original draft; JH, LU, RM: Data collection, Writing-reviewing and editing; DH. Conceptualisation, Writing-reviewing and editing; EM: Writing-reviewing and editing; SH: Conceptualisation, Methodology, Data curation, Formal analysis, Funding acquisition, Writing-original draft, and Supervision; All authors approved the version to be published and agreed to be accountable for all aspects of the work.

## Declaration of competing interest

The authors declare no conflict of interests.

## References

[1] Reid A.E., Hendricks M.K., Groenewald P., Bradshaw D. (2016). Where do children die and what are the causes? under-5 deaths in the metro west geographical service area of the Western Cape, South Africa, 2011. S Afr Med J.

[2] Troeger C., Forouzanfar M., Rao P.C., Khalil I., Brown A., Swartz S. (2017). Estimates of the global, regional, and national morbidity, mortality, and aetiologies of lower respiratory tract infections in 195 countries: a systematic analysis for the global burden of disease study 2015. Lancet Infect Dis.

[3] Ramnarayan P., Richards-Belle A., Drikite L., Saull M., Orzechowska I., Darnell R. (2022). Effect of high-flow nasal cannula therapy vs continuous positive airway pressure therapy on liberation from Respiratory support in acutely ill children admitted to pediatric critical Care units: a randomized clinical trial. JAMA.

[4] Dafydd C., Saunders B.J., Kotecha S.J., Edwards M.O. (2021). Efficacy and safety of high flow nasal oxygen for children with bronchiolitis: systematic review and meta-analysis. BMJ Open Respir Res.

[5] Maitland K., Kiguli S., Opoka R.O., Olupot-Olupot P., Engoru C., Njuguna P. (2017). Children’s oxygen administration strategies trial (COAST): A randomised controlled trial of high flow versus oxygen versus control in African children with severe pneumonia. Wellcome Open Res.

[6] Armarego M., Forde H., Wills K., Beggs S.A. High-flow nasal cannula therapy for infants with bronchiolitis - Armarego, M - 2024 Cochrane Libr.. [cited 2025 May 15]; Available from: https://www.cochranelibrary.com/cdsr/doi/10.1002/14651858.CD009609.pub3/full. 10.1002/14651858.CD009609.pub3 PMC10953464 38506440

[7] Venanzi A., Di Filippo P., Santagata C., Di Pillo S., Chiarelli F., Attanasi M. (2022). Heated humidified high-flow nasal cannula in children: state of the art. Biomedicines.

[8] Guglielmo R.D., Hotz J.C., Ross P.A., Deakers T.W., Diep J.E.L., Newth C.J.L. (2022). High-flow nasal cannula reduces effort of breathing but not consistently via positive end-expiratory pressure. Chest.

[9] Luo J.C., Lu M.S., Zhao Z.H., Jiang W., Xu B., Weng L. (2017). Positive end-expiratory pressure effect of 3 high-flow nasal cannula devices. Respir Care.

[10] Babl F.E., Franklin D., Schlapbach L.J., Oakley E., Dalziel S., Whitty J.A. (2020). Enteral hydration in high-flow therapy for infants with bronchiolitis: secondary analysis of a randomised trial. J Paediatr Child Health.

[11] Byrd C., Noelck M., Kerns E., Bryan M., Hamline M., Garber M. (2024). Multicenter quality collaborative to reduce overuse of high-flow nasal cannula in bronchiolitis. Pediatrics.

[12] Luo J., Duke T., Chisti M.J., Kepreotes E., Kalinowski V., Li J. (2019). Efficacy of high-flow nasal cannula vs standard oxygen therapy or nasal continuous positive airway pressure in children with respiratory distress: a meta-analysis. J Pediatr.

[13] Morley S.L. (2016). Non-invasive ventilation in paediatric critical care. Paediatr Respir Rev.

[14] Wilson P.T., Baiden F., Brooks J.C., Morris M.C., Giessler K., Punguyire D. (2017). Continuous positive airway pressure for children with undifferentiated respiratory distress in Ghana: an open-label, cluster, crossover trial. Lancet Glob Health.

[15] McCollum E.D., Mvalo T., Eckerle M., Smith A.G., Kondowe D., Makonokaya D. (2019). Bubble continuous positive airway pressure for children with high-risk conditions and severe pneumonia in Malawi: an open label, randomised, controlled trial. Lancet Respir Med.

[16] Gebre M., Haile K., Duke T., Faruk M.T., Kamal M., Kabir M.F. (2022). Effectiveness of bubble continuous positive airway pressure (BCPAP) for treatment of children aged 1-59 months with severe pneumonia and hypoxemia in Ethiopia: a pragmatic cluster randomized controlled clinical trial. J Clin Med.

[17] Chisti M.J., Salam M.A., Smith J.H., Ahmed T., Pietroni M.A.C., Shahunja K.M. (2015). Bubble continuous positive airway pressure for children with severe pneumonia and hypoxaemia in Bangladesh: an open, randomised controlled trial. Lancet.

[18] Mumm R., Diaz-Monsalve S., Hänselmann E., Freund J., Wirsching M., Gärtner J. (2017).

[19] Harris P.A., Taylor R., Thielke R., Payne J., Gonzalez N., Conde J.G. (2009). Research electronic data capture (REDCap)—A metadata-driven methodology and workflow process for providing translational research informatics support. J Biomed Inf.

[20] Fleming S., Thompson M., Stevens R., Heneghan C., Plüddemann A., Maconochie I. (2011). Normal ranges of heart rate and respiratory rate in children from birth to 18 years of age: a systematic review of observational studies. Lancet.

[21] World Health Organization (2014).

[22] Twomey M., Cheema B., Buys H., Cohen K., de Sa A., Louw P. (2013). Vital signs for children at triage: a multicentre validation of the revised South African Triage Scale (SATS) for children. S Afr Med J.

[23] (2019).

[24] Maitland K., Kiguli S., Olupot-Olupot P., Hamaluba M., Thomas K., Alaroker F. (2021). Randomised controlled trial of oxygen therapy and high-flow nasal therapy in African children with pneumonia. Intensive Care Med.

[25] Von Saint, André-Von Arnim A.O., Okeyo B., Cook N., Steere M., Roberts J., Howard C.R.A. (2019).

[26] Hoffman E., Reichmuth K.L., Cooke M.L. (2019). A review of the use of high-flow nasal cannula oxygen therapy in hospitalised children at a regional hospital in the Cape Town Metro, South Africa. S Afr Med J.

[27] Richards M., Le Roux D., Cooke L., Argent A. (2020). The influence of high flow nasal cannulae on the outcomes of severe Respiratory disease in children admitted to a regional hospital in South Africa. J Trop Pediatr.

[28] Schuh H.B., Hooli S., Ahmed S., King C., Roy A.D., Lufesi N. (2023). Clinical hypoxemia score for outpatient child pneumonia care lacking pulse oximetry in Africa and South Asia [Internet]. medRxiv.

